# Preparation of Perovskite-Type LaCoO_3_ and Its Catalytic Degradation of Formaldehyde in Wastewater

**DOI:** 10.3390/toxics13110955

**Published:** 2025-11-05

**Authors:** Qingguo Ma, Qin Gao, Shancheng Li, Tianying Li, Zhiqian Fan, Binglong Mu, Yike Zhang

**Affiliations:** Department of Chemistry and Chemical Engineering, Taiyuan Institute of Technology, Taiyuan 030008, China; 19934912902@163.com (Q.G.); m13594364857@163.com (S.L.); l78800104@Outlook.com (T.L.); 18233027832@163.com (Z.F.); m1375325152@163.com (B.M.); 19735107291@163.com (Y.Z.)

**Keywords:** perovskite, LaCoO_3_, wastewater, formaldehyde, degradation

## Abstract

Removing toxic formaldehyde (HCHO) from environmental water is crucial for human health and the ecosystem. Perovskite-type Lanthanum cobalt oxide (LaCoO_3_) has achieved great success in a wide range of catalytic processes; however, this concept has been rarely applied to the degradation of HCHO. Here, we prepared perovskite-type catalysts with different La/Co molar ratios, and the time for HCHO oxidation degradation at room temperature was shortened by 12 times (10 min vs. 119 min) compared to other heterogeneous catalysts. LaCoO_3_ exhibits superior catalytic activity for HCHO degradation at room temperature when the La/Co molar ratio is 1:1 compared to lanthanum cobalt oxides with other molar ratios. The X-ray photoelectron spectroscopy (XPS) test results show that increasing the La/Co molar ratio reduces the Co^2+^ content in the catalyst, while Co^2+^ plays the most important role in the catalyst. Quencher experiments indicated that sulfate radicals (SO_4_·^−^) and hydroxyl radicals (·OH) were the primary reactive species for the removal of HCHO. This finding suggests that the catalytic oxidation reaction involving HCHO operates as a heterogeneous Fenton-like oxidation reaction.

## 1. Introduction

Formaldehyde (HCHO) is a chemical raw material that has found application in various industries, including wood furniture processing, plastic synthesis, and skincare products [1,2]. Given the extensive utilization of HCHO, the inevitable consequence of its release into the environment is the production of HCHO wastewater. The World Health Organization (WHO) has classified HCHO as a suspected carcinogen and teratogen [3,4]. Degrading HCHO in wastewater is crucial for protecting aquatic environments and human health. Currently, methods for HCHO removal include adsorption [5,6], biological processes [7], photocatalysis [8], electrochemistry [9], Fenton oxidation [10], and Fenton-like oxidation [11], all widely applied for HCHO removal from wastewater.

Among these methods, adsorption is a simple and widely used method for removing HCHO from wastewater. The adsorption method has the advantages of simple adsorption material structure, convenient operation and high adsorption efficiency. There are many kinds of adsorbents, such as porous carbon [12,13], polymers [14], nano-powders [15], etc. These adsorption materials all have a good pore structure, high specific surface area, good thermal stability and chemical resistance to alkaline and acidic media. However, the adsorption process does not involve degradation, the adsorbed pollutants still exist and may continue to cause pollution. Given that the adsorption of HCHO occurs exclusively from wastewater to the adsorbent, with no subsequent breakdown into harmless environmental substances, the HCHO accumulated in the adsorbent remains a concern. Furthermore, the adsorption capacity of adsorbents is not unlimited. In most cases, adsorbents eventually reach a state of saturation, which leads to desorption. Consequently, alternative degradation methods currently under development for the removal of HCHO from wastewater include biological processes and advanced oxidation processes (AOPs). These methodologies have been demonstrated to achieve the mineralization of HCHO. In particular, biological methods have been shown to be more environmentally friendly, with a negligible propensity for secondary pollution. Nevertheless, the utilization of microorganisms in biotechnology necessitates prolonged domestication and screening processes. A prevalent challenge pertains to the microorganisms’ limited tolerance for HCHO wastewater, which often results in a low survival rate in high-concentration HCHO wastewater or acid–base environments [16]. AOPs are highly regarded due to their broad applicability, strong anti-interference capabilities, and the degradation of organic matter through mineralization. The photocatalytic method, electrochemical method, Fenton oxidation method, and Fenton-like oxidation method can all be considered as AOPs, and the goal of these methods is to generate highly oxidizing free radicals capable of mineralizing pollutants. The key factors in the process of treating organic pollutants by photocatalytic method, electrochemical method, Fenton oxidation method, and Fenton-like oxidation method are the generation of reactive species [17]. The rate of degradation of organic pollutants is contingent upon the number of free radicals produced and their molecular structure [18]. Photocatalysis suffers from low degradation efficiency, mainly due to the large difference in the absorption effect of the reaction system on light, and under high-power light radiation, the number of active free radicals produced is limited [19,20].

The hydroxyl radicals produced by the electrochemical method are predominantly concentrated in the proximity of the electrode. The probability of these radicals colliding and reacting with the HCHO molecules dispersed in the solution system is minimal due to the remarkably brief survival duration of the hydroxyl radicals (10^−6^–10^−3^ s) [21]. Fenton oxidation is defined as a specific oxidation system composed of H_2_O_2_, Fe^2+^, and acid. This system facilitates the decomposition of hydrogen peroxide through Fe^2+^, resulting in the production of hydroxyl radicals (·OH). These hydroxyl radicals subsequently attack and mineralize organic pollutants, thereby achieving deep oxidation of organic pollutants. Fenton oxidation boasts a number of advantages, including its rapid reaction rate and high organic removal rate, as well as its low energy requirements [14]. This makes it a widely used method for the treatment of organic pollutants in industrial wastewater [22,23]. For example, Fenton oxidation was employed to treat Octahydro-1,3,5,7-tetranitro-1,3,5,7-tetrazocine (HMX) wastewater. The results show that under the reaction conditions of pH = 3.0, H_2_O_2_ concentration of 30.0 mmol/L, and Fe^2+^ concentration of 0.7 mmol/L, the degradation performance of HMX reached its optimum after 180 min, with a removal rate as high as 90.8% [24]. Caiyi Jiang prepared Fe–N co-doped carbon catalyst (αFe@CTS-T) and used it to degrade RhB. αFe@CTS-T releases Fe^2+^ during the reaction process, which activates hydrogen peroxide to generate hydroxyl radicals. The maximum degradation rate of RhB was 99%. High catalytic activity was attributed to the high content of Fe^2+^ and Fe3^+^ in the catalyst [25]. Although the H_2_O_2_, Fe^2+^, and acid system is effective in degrading organic pollutants, this process also has disadvantages. As the reaction proceeds, Fe^2+^ will convert into Fe^3+^, and then form iron precipitation (Fe(OH)_3_), which affects the activation ability of hydrogen peroxide, and ultimately leads to the cessation of the oxidation reaction. In order to sustain the degradation reaction, it is imperative to add Fe^2+^ continuously, so this process consumes a large amount of catalyst Fe^2+^, and a secondary pollutant, iron mud (Fe(OH)_3_), was generated. Moreover, the Fenton oxidation process needs to be carried out under strong acid conditions to ensure a high degradation rate. Finally, when degrading high-concentration HCHO wastewater, the degradation efficiency is not ideal [26].

The heterogeneous Fenton catalyst has been demonstrated to exhibit characteristics such as high recyclability and ease of operation. In addition, it has been observed to reduce the amount of catalyst required, yet its catalytic activity is lower than that of the homogeneous Fenton catalyst [27,28,29]. A heterogeneous Fenton catalyst GF-PDA-FeOOH was prepared with glass fiber as the carrier, and glass fiber was modified through oxidative self-polymerization of dopamine, and then prepared by the impregnation method in an FeSO_4_ solution. Although the GF-PDA-FeOOH catalyst exhibits excellent cyclic performance, the degradation rate of methylene blue (MB), Congo red (CR) and crystal violet (CV) can still maintain above 80% after being used 6 cycles, When the degradation rates of MB, CR, and CV exceed 90%, the required temperature is 40 °C, and the time exceeds 100 min [30]. In order to improve the oxidation efficiency of the heterogeneous Fenton-like oxidation reaction, researchers introduced light to synergistically catalyze the formation of free radicals. Athikaphan P et al. [31] prepared a catalyst n-ZVI/TiO_2_ by wet impregnation and sodium borohydride reduction method. The degradation of HCHO was only 55.07% without UV light. The complete degradation of HCHO can only be achieved by adding the catalyst and then exposing it to UV light. Moreover, the degradation of HCHO under this catalyst condition is greatly affected by pH value. The degradation efficiency of HCHO rapidly decreased when pH value was greater than 3 because the catalyst can only catalyze the generation of hydroxyl radicals from hydrogen peroxide under acidic conditions. Bi F et al. [32] successfully prepared a new type of magnetic recyclable ZnO/NiFe_2_O_4_ composite nanofibrous photocatalyst by parallel electrospinning. When ZnO/NiFe_2_O_4_ is used as a catalyst, 0.05 mL of H_2_O_2_ is added to the reaction system and the degradation rate of RhB can reach 99.57% after 10 min of illumination. Without light irradiation, the degradation rate of RhB was only 10% after reacting in the dark for 30 min. In the absence of hydrogen peroxide and only with catalyst ZnO/NiFe_2_O_4_, the degradation rate of RhB was only 50.99% after 210 min of reaction. The degradation reaction is promoted in the presence of light irradiation. However, the implementation of light irradiation has been observed to result in an increase in the establishment and operational costs of the Fenton-like oxidation method. It is imperative to identify catalysts capable of facilitating the conversion of hydrogen peroxide and potassium peroxymonosulfate into ·OH and SO_4_·^−^ during oxidation processes. The method of introducing transition metal-activated oxidants to generate free radicals has been demonstrated to be more economical and easier to operate than light irradiation [33].

Perovskite catalysts have been extensively studied in the catalytic treatment of wastewater and have been shown to have good degradation effects [34,35,36]. The chemical general formula of perovskite-type oxides is ABO_3_. Lanthanide-based perovskite has been reported to be a good candidate for heterogeneous catalysis. In addition, Co is the most effective transition metal for homogeneous catalysis, so heterogeneous Co-based catalysts exhibiting low Co^2+^ leaching rates have attracted the attention of researchers. Therefore, it is necessary to introduce Co into the B site of perovskite catalysts to prepare stable cobalt-containing perovskite-type catalysts [37].

Although the perovskite-type lanthanum cobalt oxide has been extensively studied in catalytic oxidation, its application in the degradation of HCHO has not been reported. Additionally, there is no literature on the effect of the La/Co molar ratio on the catalytic activity. In this study, we have prepared perovskite-type lanthanum cobalt oxide nanoparticle catalysts with varying La/Co molar ratios via the sol–gel method. A structural analysis of lanthanum cobalt oxides prepared with varying La/Co molar ratios reveals that they all exhibit a perovskite structure. However, their catalytic activity varies during HCHO degradation. The lanthanum cobalt oxides with a La/Co molar ratio of 1:1 exhibited the highest catalytic activity for HCHO degradation. As the La/Co molar ratio increased, the catalytic activity for HCHO degradation decreased. We investigated the effect of different process parameters (catalyst type, catalyst concentration, oxidant type, oxidant concentration, HCHO concentration, initial pH value) on the degradation efficiency of HCHO, and to determine the optimal experimental conditions. Our research found that although perovskite-type lanthanum cobalt oxides exhibit excellent catalytic performance in promoting PMS-mediated HCHO degradation, their efficacy in catalyzing hydrogen peroxide-mediated HCHO degradation is unsatisfactory.

## 2. Materials and Methods

### 2.1. Materials

Lanthanum nitrate (La(NO_3_)_3_·6H_2_O) with a purity of 99.5%, cobalt nitrate (Co(NO_3_)_2_·6H_2_O) with a purity of 98.5%, HCHO solution (37 wt%), sodium thiosulfate (Na_2_S_2_O_3_) with a purity of 99.0%, Potassium monopersulfate salt (KHSO_5_) with a purity of 42.0%, acetic acid (CH_3_COOH) with a purity of 99.5% and acetylacetone (CH_3_COCH_2_COCH_3_) with a purity of 99.0%. They were all purchased from Sinopharm Chemical Reagent Co., Ltd., Shanghai, China.

### 2.2. Methods

#### 2.2.1. Preparation of Lanthanum Cobalt Oxide

The required reagent amounts were calculated based on a molar ratio of lanthanum nitrate, cobalt nitrate, and citric acid of 1:1:4. 3.523 g of lanthanum nitrate and 2.368 g of cobalt nitrate were weighed separately and dissolved in beakers each containing 5 mL of deionized water to prepare the corresponding solutions. Under stirring conditions, the lanthanum nitrate and cobalt nitrate solutions were combined. 6.252 g of citric acid was added slowly to this mixed solution, and after complete dissolution, the mixed solution was heated to 80 °C and the reaction is continued until the mixed solution is converted into a viscous sol. The gel was transferred to a drying oven and dried at 120 °C for 6 h. Finally, the dried solid was transferred to a crucible and calcined in a muffle furnace at 600 °C for 4 h to obtain a solid powder, denoted as LaCoO_3_-1. The molar ratios of lanthanum nitrate, cobalt nitrate, and citric acid were adjusted to 2:1:6 and 3:1:8, respectively. Lanthanum cobalt oxide was prepared following the same method as above and designated as LaCoO_3_-2 and LaCoO_3_-3.

#### 2.2.2. Characterization of Lanthanum Cobalt Oxide

Thermogravimetric (TG, PerkinElmer, TGA4000, Waltham, MA, USA) analysis was performed on lanthanum cobalt oxides and their precursors. Samples were tested under an atmosphere with a flow rate of 20 mL/min. The temperature range was 50–800 °C, with a heating rate of 10 °C/min. The sample was stabilized at 100 °C for 5 min during the heating process.

The lanthanum cobalt oxide of LaCoO_3_-1, LaCoO_3_-2, and LaCoO_3_-3 was analyzed using X-ray powder diffraction (XRD, Rigaku, Smartlab, Tokyo, Japan) (copper target radiation source, step size 0.02°, 2θ = 5–80°).

Transmission electron microscopy (TEM, JEOL, JEM-F200, Tokyo, Japan) analyzed the grain size and interplanar spacing of LaCoO_3_-1.

The valence states of lanthanum and cobalt in LaCoO_3_-1 were analyzed using X-ray photoelectron spectroscopy (XPS, Thermo Fischer, ESCALAB 250Xi, Waltham, MA, USA). Cobalt measurement conditions: Al Kα radiation, total acquisition time 308.1 s, test energy 30.0 eV, step size 0.10 eV, 411 steps. Lanthanum testing conditions: Al Kα radiation, total acquisition time 200.4 s, test energy range 30.0 eV, step size 0.10 eV, 401 steps.

#### 2.2.3. The Performance of HCHO Degradation

HCHO solution (approximately 1 mg/mL), sodium hydroxide solution, and iodine solution were added to the iodine bottle. After thoroughly mixing, sulfuric acid solution was added. The reaction was allowed to proceed for 15 min, followed by titration with standard sodium thiosulfate solution. A blank test was performed by replacing HCHO solution with deionized water and titrating with standard sodium thiosulfate solution using the same method. The concentration of the HCHO standard solution was calculated based on the volume of standard sodium thiosulfate solution consumed, resulting in 1.086 mg/mL [38].

5 mL of HCHO standard solution was added to a 10 mL reaction tube. After the temperature of the HCHO solution stabilized at 25 °C, 0.06 g of LaCoO_3_ and 0.13 g of PMS were added. The reaction was then started and allowed to proceed for 10 min. Every 2 min, 30 μL of reaction solution sample was taken, filtered through a filter membrane with a pore size of 0.45 μm, and reacted with sodium thiosulfate solution to terminate the reaction. The resulting solution was transferred to a colorimetric tube, followed by the addition of acetic acid and acetylacetone. Finally, the solution was diluted to the mark with deionized water. The colorimetric tubes were transferred to a boiling water bath to react for 3 min. The concentration of HCHO in the solution was determined by visible spectrophotometry. Finally, the degradation rate of HCHO was calculated based on the concentration of HCHO [38]. The effect of different process parameters (catalyst type, catalyst concentration, oxidant type, oxidant concentration, HCHO concentration, initial pH value) on the degradation efficiency of HCHO was investigated.

## 3. Results

### 3.1. TG Analysis

**Figure 1:**
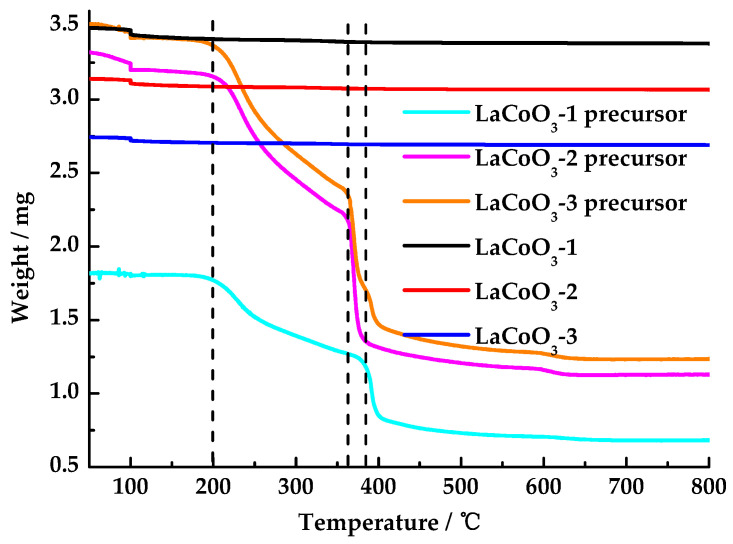
TG curves of LaCoO_3_-1, LaCoO_3_-2, LaCoO_3_-3, LaCoO_3_-1 precursor, LaCoO_3_-2 precursor, and LaCoO_3_-3 precursor.

As shown in Figure 1, lanthanum cobalt oxide precursors showed three stages of weight loss. The first stage started at 200 °C, which was due to the decomposition of unreacted citric acid. Within 360(380)–400 °C, lanthanum cobalt oxide precursors underwent rapid weight loss, primarily due to the decomposition of citrates and nitrates. The decomposition temperature of LaCoO_3_-2 precursor and LaCoO_3_-3 precursor in the second stage was 20 °C lower than that of LaCoO_3_-1 precursor. This difference may be attributed to the observation that with an increase in the La/Co molar ratio, the decomposition temperatures of citrate and nitrate decrease. Lanthanum cobalt oxide precursors underwent a gradual weight loss within the temperature range of 400–600 °C, a phenomenon primarily attributed to the decomposition of residual citrate and nitrate. Above 600 °C, no significant weight loss was observed, indicating that LaCoO_3_ was formed at 600 °C. The solid powders obtained by calcining the lanthanum cobalt oxide precursor at 600 °C, namely LaCoO_3_-1, LaCoO_3_-2, and LaCoO_3_-3, exhibited almost no weight loss in the temperature range of 50–800 °C, which also indicates that the lanthanum cobalt oxide was formed at 600 °C. Consequently, 600 °C was selected as the final calcination temperature.

### 3.2. XRD Analysis

**Figure 2:**
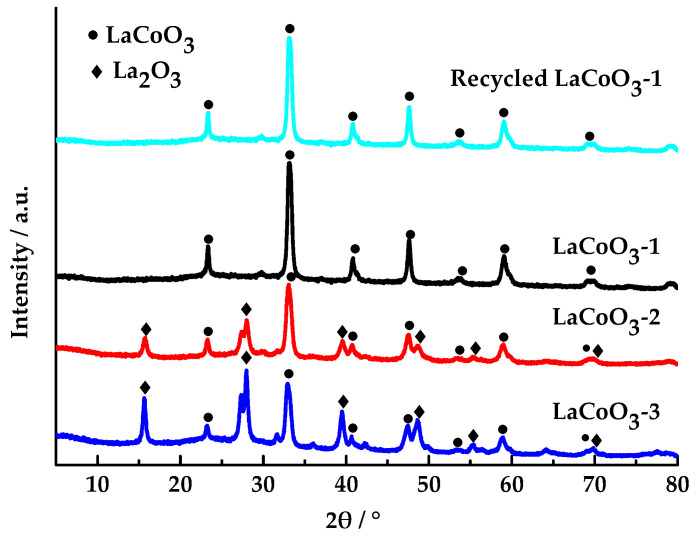
XRD patterns of LaCoO_3_-1, LaCoO_3_-2, LaCoO_3_-3, and recycled LaCoO_3_-1.

As shown in Figure 2, the diffraction peaks (23.22°, 32.88°, 40.66°, 47.46°, 53.6°, 58.92°, 69.82°, and 78.76°) correspond to those of perovskite-type LaCoO_3_ (JCPDS No. 48-0123) [36,39], and the diffraction peaks (15.72°, 27.96°, 39.58°, 48.58°, 55.36°, 64.3°, and 69.84°) correspond to those of La_2_O_3_ (JCPDS No. 02-0607) [40]. All the samples possess perovskite-structured LaCoO_3_ phases. No other peaks were detected except for the perovskite phase, indicating that the LaCoO_3_-1 has a single phase. In contrast, LaCoO_3_-2 and LaCoO_3_-3 contain both the perovskite phase and the La_2_O_3_ phase, indicating that the materials are two-phase. As the molar ratio of La/Co increases, the diffraction intensity of the perovskite-type LaCoO_3_ decreases, and a crystalline phase of La_2_O_3_ appears, whose diffraction intensity also increases with the molar ratio of La/Co. This indicates that when the molar ratio of La/Co is 1:1, a perovskite structure of La-O-Co is formed. As the molar ratio of La/Co increases, there is an excess of La atoms. The surplus La atoms form a La-O-La structure, resulting in the appearance of La_2_O_3_ crystalline phase in the spectrum. The XRD pattern of the recycled LaCoO_3_-1 indicates that the perovskite-type remains unaltered, suggesting that the perovskite-type LaCoO_3_-1 exhibits remarkable reusability.

### 3.3. TEM Analysis

**Figure 3:**
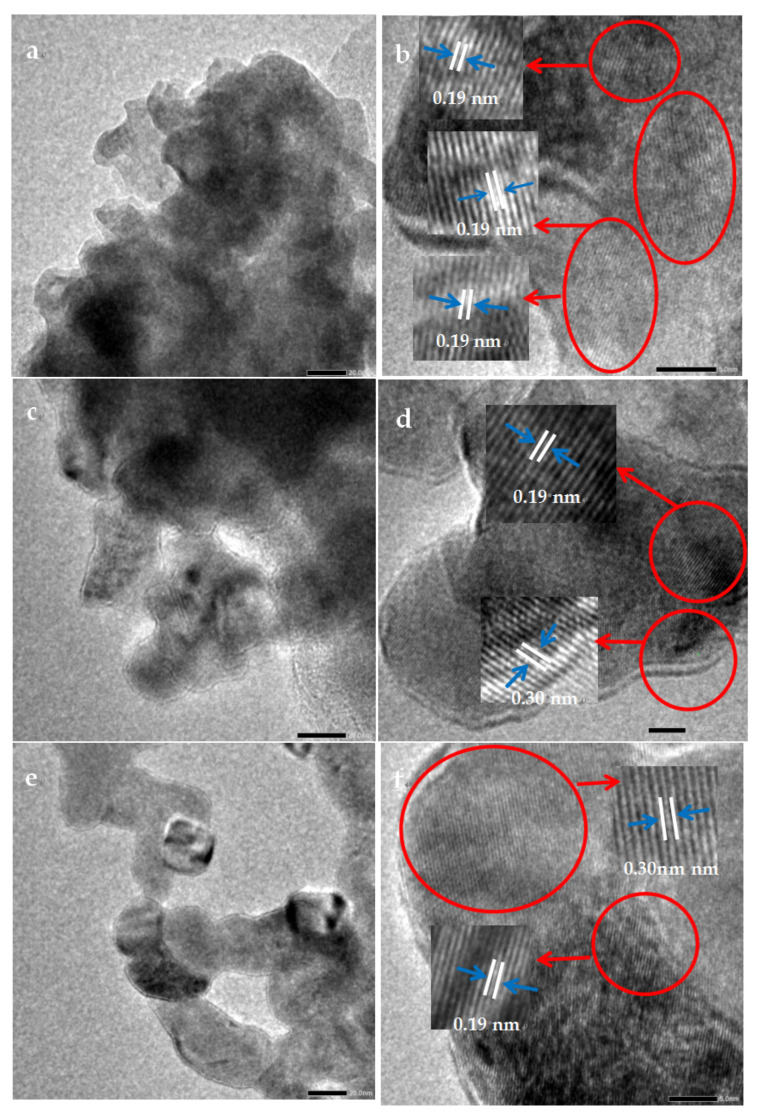
TEM images of (**a**) LaCoO_3_-1; (**c**) LaCoO_3_-2; (**e**) LaCoO_3_-3 and HRTEM images of (**b**) LaCoO_3_-1; (**d**) LaCoO_3_-2; (**f**) LaCoO_3_-3.

As shown in Figure 3, the particle sizes of the LaCoO_3_-1, LaCoO_3_-2, and LaCoO_3_-3 all range from 20 to 50 nm. The particles of LaCoO_3_-3 are more uniformly dispersed than those of LaCoO_3_-1 and LaCoO_3_-2. The lattice fringe spacings of 0.19 nm and 0.30 nm correspond well to the (024) crystal plane of perovskite-structured LaCoO_3_ and the (002) crystal plane of La_2_O_3_, respectively [41]. LaCoO_3_-1 exhibited only the (024) crystal plane, while LaCoO_3_-2 and LaCoO_3_-3 displayed both crystal planes, indicating that both perovskite and lanthanum oxide crystal structures coexist in LaCoO_3_-2 and LaCoO_3_-3.

### 3.4. XPS Analysis

**Figure 4:**
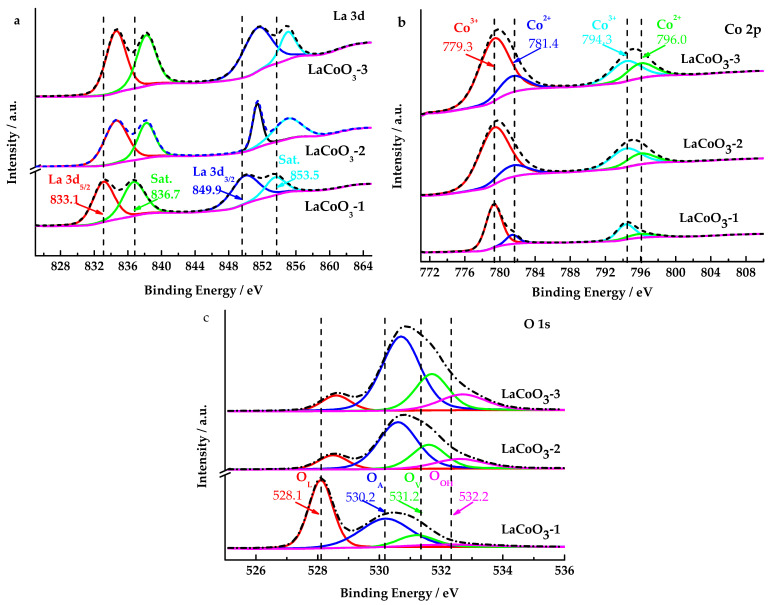
XPS spectra of LaCoO_3_-1, LaCoO_3_-2 and LaCoO_3_-3 ((**a**): La 3d; (**b**): Co 2p; (**c**): O 1s).

Figure 4a shows the La 3d spectra of LaCoO_3_-1, LaCoO_3_-2 and LaCoO_3_-3, which all contained four peaks. The La3d XPS spectrum of LaCoO_3_-1 reveals a binding energy of 849.9 eV for La 3d_3/2_ and 833.1 eV for La 3d_5/2_, with satellite peaks appearing at 853.5 eV and 836.7 eV. The La 3d splitting energy is about 16.8 eV, close to the splitting energy of pure La_2_O_3_, indicating that the oxidation state of lanthanum element species in LaCoO_3_-1 is primarily +3 [42,43]. Since LaCoO_3_-2 and LaCoO_3_-3 have increased the La_2_O_3_ content, the electron binding energy of La 3d in LaCoO_3_-2 and LaCoO_3_-3 has increased.

As shown in Figure 4b, the Co 2p XPS spectrum of the LaCoO_3_-1, LaCoO_3_-2 and LaCoO_3_-3 could be fitted into four peaks. The two peaks located at 779.3 and 794.3 eV were close to the standard data for Co^3+^, while the other two peaks at 781.4 eV and 796.0 eV were related to Co^2+^ [43]. Although the four peaks of the three samples were at the same position, the proportion of Co^2+^ in the total Co was different. The proportions of Co^2+^ to total Co atom in the LaCoO_3_-1, LaCoO_3_-2 and LaCoO_3_-3 are 19.07%, 22.13%, and 25.25%, respectively. However, as the La/Co molar ratio increased, the content of Co atoms in the sample decreased. XPS data indicates that the Co at% proportion in LaCoO_3_-1, LaCoO_3_-2, and LaCoO_3_-3 are 10.39, 4.58, and 3.60, respectively, and the La at% proportion are 17.72, 19.82, and 19.16, respectively. Consequently, the atomic ratios of La atoms to Co atoms in LaCoO_3_-1, LaCoO_3_-2, and LaCoO_3_-3 are determined to be 1.7, 4.3, and 5.3, respectively. The molar ratios of La to Co in the raw materials used for preparing LaCoO_3_-1, LaCoO_3_-2, and LaCoO_3_-3 are 1, 2, and 3, respectively. Therefore, it can be deduced that the atomic ratios of La atoms to Co atoms in LaCoO_3_-1, LaCoO_3_-2, and LaCoO_3_-3 are 1, 2, and 3, respectively. As indicated by the aforementioned data, an increase in the molar ratio of La to Co results in an increased probability of La atoms being distributed on the catalyst surface.

Figure 4c displays that four oxygen species at 528.1, 530.2, 531.2 and 532.2 eV were observed in the O 1s spectra of LaCoO_3_-1, which were assigned to the lattice oxygen of metal oxides (O^2−^, denoted as O_L_), adsorbed oxygen species (O_2_^2−^O^−^, denoted as O_A_), oxygen vacancy (O_v_), and surface hydroxyl species (OH^−^, denoted as O_OH_), respectively [44].

Compared to LaCoO_3_-1, the electron binding energy of O 1s and La 3d in LaCoO_3_-2 and LaCoO_3_-3 has increased. The proportions of O_L_ to total O atoms decrease sequentially in LaCoO_3_-1, LaCoO_3_-2 and LaCoO_3_-3, with proportions of 43.89%, 11.96%, and 8.83%, respectively. These results suggest that the increase in lanthanum oxide can promote the reduction of lattice oxygen, which is detrimental to catalytic oxidation reactions.

### 3.5. Investigation of Reaction Conditions for HCHO Degradation

#### 3.5.1. Effects of Catalyst Types on HCHO Degradation

**Figure 5:**
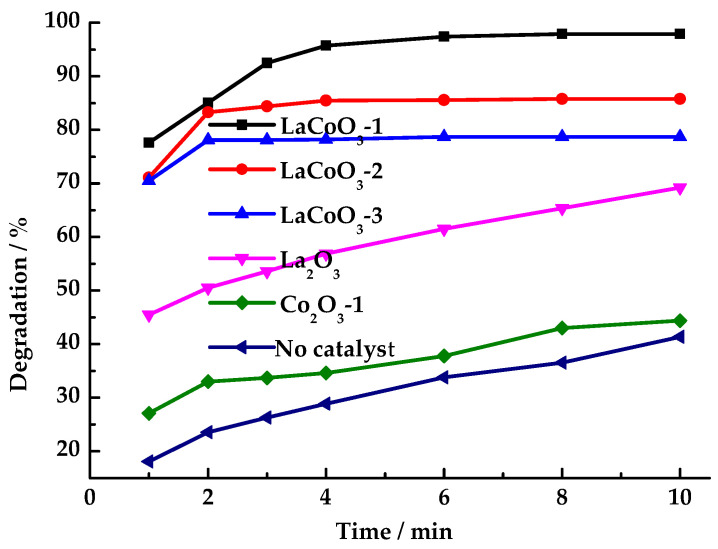
Effects of catalyst types on degradation rate of HCHO (Reaction conditions: HCHO solution 1.086 mg/mL, PMS 26 mg/mL, catalyst 12 mg/mL, 25 °C).

As shown in Figure 5, the HCHO degradation performance of LaCoO_3_ catalysts with different La/Co molar ratios was investigated. The study revealed that without a catalyst, the HCHO degradation rate reached only 40.1% after 10 min. The perovskite-type LaCoO_3_-1 exhibited the best catalytic effect, achieving a HCHO degradation rate of 95.7% within 4 min. As the lanthanum content increased, the HCHO degradation rate decreased significantly. When using LaCoO_3_-2 and LaCoO_3_-3 as catalysts for HCHO degradation, the degradation rates were 85.8% and 78.7%, respectively. The degradation of HCHO slowed after 2 min. When catalysts LaCoO_3_-1, LaCoO_3_-2 and LaCoO_3_-3 were used, respectively, the degradation rate of HCHO increased by only 2%, 3%, and 4% after 2 min. Under conditions where only catalysts LaCoO_3_-1, LaCoO_3_-2, LaCoO_3_-3 without PMS as the oxidant, HCHO degradation was negligible. Moreover, the removal rates of HCHO were not significantly different among the three types of catalysts (less than 5%), indicating that the prepared LaCoO_3_ catalyst exhibits weak adsorption capacity for HCHO.

Although the HCHO degradation performance of LaCoO_3_-1 is higher than LaCoO_3_-2 and LaCoO_3_-3, LaCoO_3_-2 and LaCoO_3_-3 exhibited greater catalytic activity toward PMS than LaCoO_3_-1 in the reaction system. Testing with starch-potassium iodide paper revealed that the reaction solution no longer turned the paper blue after 8 min, indicating that LaCoO_3_-2 and LaCoO_3_-3 catalyzed the production of SO_4_·^−^ and ·OH from PMS at a faster rate. However, these species underwent quenching on the catalyst surface and did not fully participate in the HCHO oxidation reaction. Furthermore, the PMS concentration dropped to a low level after 2 min, reducing the efficiency of continued HCHO degradation. LaCoO_3_-1 catalyzed PMS to produce SO_4_·^−^ and ·OH at a slower rate than LaCoO_3_-2 and LaCoO_3_-3. Moreover, the residence time of SO_4_·^−^ and ·OH in the reaction system catalyzed by LaCoO_3_-1 is longer than that in systems catalyzed by LaCoO_3_-2 and LaCoO_3_-3, thus demonstrating higher catalytic efficiency than LaCoO_3_-2 and LaCoO_3_-3.

This phenomenon can be attributed to the fact that an increase in La content in LaCoO_3_ leads to enhanced adsorption of PMS on the surface of LaCoO_3_, thereby augmenting the catalytic activity of LaCoO_3_ for PMS. This, in turn, results in the acceleration of the rate of free radical generation by PMS, with a consequent quenching of a substantial number of active free radicals on the surface of LaCoO_3_.

When La_2_O_3_ and Co_2_O_3_ were used as catalysts, the degradation rates of HCHO were 69.2% and 44.4%, respectively. The reaction solution was detected by starch-potassium iodide paper, which was still blue after 120 min. This finding indicates that the presence of mixed Co^3+^/Co^2+^ oxidation states within the LaCoO_3_ perovskite structure is a significant contributing factor. Furthermore, the catalytic mechanism of LaCoO_3_ deviates from that of La_2_O_3_ and Co_2_O_3_. The instability of the O-O bond in PMS, which is prone to break after receiving electrons, is a key factor in this process. The electron transfer between Co^2+^ and Co^3+^ in LaCoO_3_ promotes the cleavage of O-O and generates SO_4_·^−^ [45]. Subsequent studies selected LaCoO_3_-1 as the catalyst to investigate its catalytic performance in degrading HCHO by PMS.

**Figure 6:**
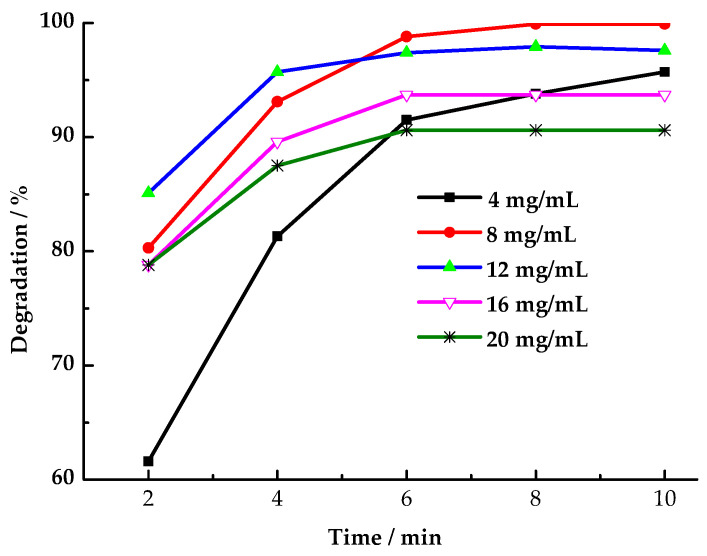
Effect of LaCoO_3_-1 dosage on degradation rate of HCHO (Reaction conditions: HCHO solution 1.086 mg/mL, PMS 26 mg/mL, 25 °C).

As shown in Figure 6, the HCHO degradation rate peaked at 99.9% after 8 min when LaCoO_3_-1 was used at a dosage of 8 mg/mL. At a LaCoO_3_-1 dosage of 12 mg/mL, the HCHO degradation rate exceeded that of other dosages during the initial 4 min. However, after a period of 6 min, the observed degradation rate fell below that which was recorded at the 8 mg/mL dosage. This finding suggests that when the LaCoO_3_-1 dosage is less than 12 mg/mL, increasing the dosage accelerates the rate at which LaCoO_3_-1 activates PMS to produce SO_4_·^−^ and ·OH, thereby enhancing HCHO degradation efficiency. However, as time progresses, the PMS concentration decreases, reducing the generation rate of SO_4_·^−^ and ·OH and consequently lowering the HCHO degradation efficiency. When the LaCoO_3_-1 dosage exceeds1 2 mg/mL, LaCoO_3_-1 catalyzed PMS to produce SO_4_·^−^ and ·OH more rapidly. Some SO_4_·^−^ and ·OH fail to react with HCHO molecules before undergoing quenching [46]. In other words, the self-quenching reaction between the excess free radicals leads to a decrease in the HCHO degradation rate.

#### 3.5.2. Effect of PMS Dosage on HCHO Degradation

**Figure 7:**
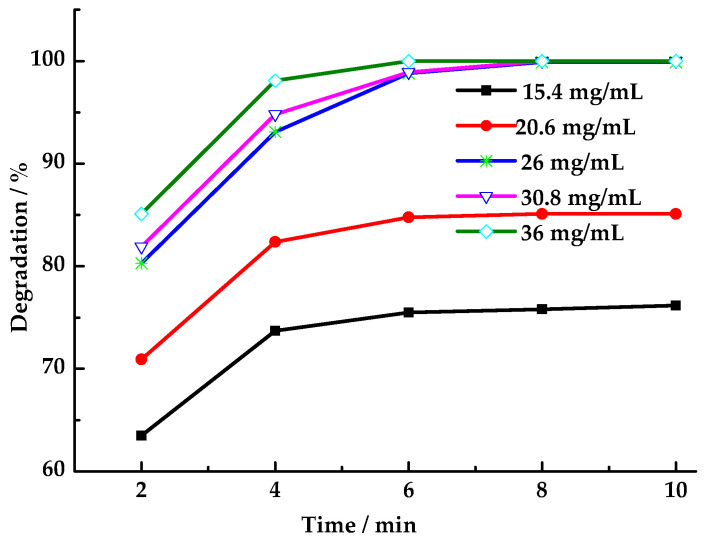
Effect of PMS dosage on degradation rate of HCHO (Reaction conditions: HCHO solution 1.086 mg/mL, catalyst 8 mg/mL, 25 °C).

As shown in Figure 7, the HCHO degradation rate increases with the PMS dosage. This phenomenon can be attributed to the observation that increasing the dosage of PMS results in an elevation of the concentration of SO_4_·^−^ and ·OH generated by LaCoO_3_-1 catalysis within the reaction system. Consequently, this increase in concentration leads to an enhancement in the probability of interaction between SO_4_·^−^ and ·OH with HCHO molecules. After 6 min, the concentration of PMS in the reaction system becomes negligible, leading to the imminent cessation of the HCHO degradation reaction. It has been demonstrated that, at a constant PMS dosage, an extension of the reaction time beyond 6 min has a negligible effect on the degradation rate. When the oxidant PMS was replaced with an equivalent molar amount of H_2_O_2_ (43 μL) while maintaining other reaction conditions, the HCHO degradation rates at 2, 4, 6, 8, and 10 min were 14.5%, 27.6%, 30.1%, 30.8%, and 30.8%, respectively. These degradation rates were found to be considerably lower than those observed with PMS as the oxidant. Given the comparable oxidative capabilities of SO_4_·^−^ and ·OH, the significant disparity in degradation rates suggests one of two possibilities. The catalyst LaCoO_3_-1 may exhibit no catalytic activity toward hydrogen peroxide, or the ·OH generated by the catalyst may not participate in the oxidation reaction for HCHO degradation. Monitoring hydrogen peroxide concentration changes in the solution using starch-potassium iodide paper revealed no hydrogen peroxide residue after 6 min. This indicates that LaCoO_3_-1 catalyzed the generation of ·OH and ·OOH from hydrogen peroxide [47]. However, these radicals persisted in the reaction system for an insufficient duration to participate in the oxidation of HCHO. Instead, they combined on the catalyst surface, forming water (lacking oxidizing capacity) and oxygen (with weaker oxidizing capacity), which rapidly escaped from the reaction system. This also demonstrates that SO_4_·^−^ exhibits a longer lifetime than ·OH in this reaction system [48]. The long existence time of SO_4_·^−^ in the reaction system increases its contact opportunity with HCHO molecules, thus improving the degradation rate.

#### 3.5.3. Effect of HCHO Concentration on HCHO Degradation

**Figure 8:**
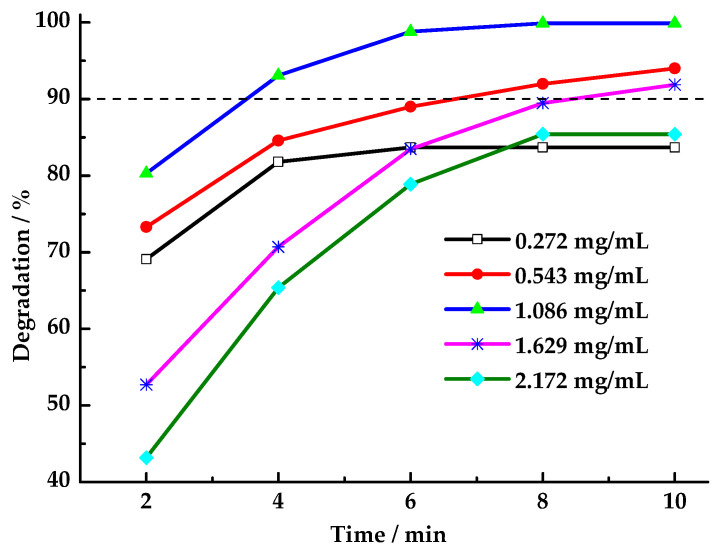
Effect of HCHO concentration on degradation rate of HCHO (Reaction conditions: n(PMS)/n(HCHO) = 2.5, catalyst 8 mg/mL, 25 °C).

The applicable HCHO concentration of the catalyst and oxidant was investigated by changing the HCHO concentration with an optimized amount of catalyst and oxidant. As shown in Figure 8, when the HCHO concentration was 0.543 mg/mL, 1.086 mg/mL, 1.629 mg/mL, the degradation rates of HCHO were all above 90%. Consequently, at a constant catalyst concentration and molar ratio of PMS/HCHO, a decrease in HCHO concentration results in a reduction in PMS concentration within the system. While this enhancement of the catalyst’s efficiency in activating PMS to generate radicals is advantageous, it concomitantly diminishes the contact opportunities between PMS and HCHO, resulting in lower degradation rates. Conversely, elevated HCHO concentrations have been observed to concomitantly increase PMS concentrations within the system. However, when the amount of catalyst remains constant, the catalyst’s capacity to catalyze PMS activation and generate free radicals is limited, resulting in incomplete HCHO degradation. Consequently, to attain elevated HCHO degradation rates across a broad concentration range, the quantity of both the catalyst and oxidant in the reaction system must be adjusted concurrently.

#### 3.5.4. Effect of pH Value on HCHO Degradation

**Figure 9:**
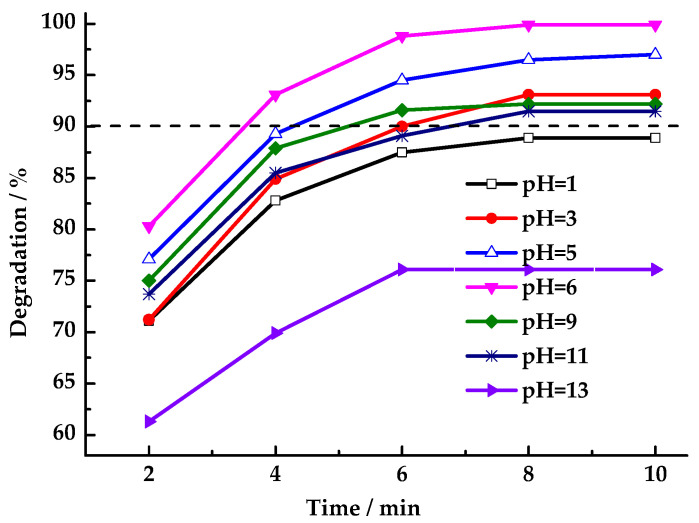
Effect of pH value on degradation rate of HCHO (Reaction conditions: n(PMS)/n(HCHO) = 2.5, catalyst 8 mg/mL, 25 °C).

The acidity or alkalinity of HCHO wastewater is uncertain, and the pH value of the solution will affect the removal efficiency of pollutants. Thus, the catalytic oxidation system must perform effectively across a broad pH range. As shown in Figure 9, the performance of LaCoO_3_-1 in activating PMS for HCHO degradation was investigated across a range of solutions with different pH values. Results indicate that under near-neutral conditions (pH = 3–11), HCHO degradation rates exceeded 90% within 10 min, with neutral conditions yielding the highest degradation rate. As acidity or alkalinity increased, the HCHO degradation rate decreased. The presence of strong acids and bases in the reaction medium was found to be deleterious to the degradation process. Under alkaline conditions, the degradation rate declined because SO_4_·^−^ reacted with OH^−^ to form hydroxyl radicals (·OH), which have a relatively short lifetime in the solution system. Under acidic conditions, pH changes may cause varying amounts of lanthanum or cobalt ions to leach into the solution, disrupting the catalyst structure. Overall, LaCoO_3_-1-catalyzed PMS degradation of HCHO exhibits a broad pH tolerance range.

#### 3.5.5. Reusability and Stability of the Perovskite LaCoO_3_-1

LaCoO_3_-1 was subjected to four repeated catalytic degradation experiments of HCHO, and the degradation rates of HCHO were 98%, 96%, 94%, and 91%, respectively. After four cycles, the degradation rate remains above 90%, and the XRD pattern of the recycled LaCoO_3_-1 shows that the perovskite structure has not changed, indicating that the perovskite LaCoO_3_-1 has excellent reusability. The gradual decrease in degradation rate may result from minor loss of active components within the catalyst. Since the highest stable oxidation state of cobalt is +3, it is possible that part of Co^2+^ in the LaCoO_3_-1 is converted to Co^3+^ as the catalyst is used, thus the catalytic efficiency is reduced [49].

## 4. Discussion

To further investigate the HCHO degradation mechanism in the LaCoO_3_-1/PMS system. A series of experiments were conducted with the use of scavengers, the objective of which was to ascertain whether the reaction in question was of a radical nature. Tert-butanol was considered in order to determine the contribution of radicals in the degradation of HCHO. After adding t-butanol (1 mL), the HCHO degradation efficiency decreased significantly (67.3%), whereas adding an equal volume of deionized water had almost no effect on the HCHO degradation efficiency (98.7%). A plausible mechanism is proposed to explain the catalytic degradation of HCHO by LaCoO_3_-1 and PMS system. The LaCoO_3_-1 continuously activates PMS through reversible redox reactions of Co^2+^/Co^3+^, efficiently producing SO_4_·^−^ and ·OH, and acting on HCHO. PMS was adsorbed onto the LaCoO_3_-1 surface for reaction, but it also accelerated electron transfer and promoted Co^2+^ regeneration. The initial reaction of Co^2+^ and PMS results in the formation of SO_4_·^−^, OH^−^ and Co^3+^, with the concomitant reduction of Co^3+^ to Co^2+^. Furthermore, SO_4_·^−^and OH^−^ can react to form ·OH, thus forming a Co^2+^/Co^3+^ cycle, ensuring sufficient Co^2+^ to continuously catalyze PMS during HCHO degradation and improving degradation efficiency.

Perovskite-type LaCoO_3_-1 has been shown to catalyze the PMS reaction, thereby generating radicals. In addition, it has been observed to catalyze the decomposition of hydrogen peroxide. However, they cannot catalyze the oxidative degradation of HCHO by hydrogen peroxide. A substantial advancement would be achieved by adjusting the perovskite structure and elemental composition to achieve catalytic oxidative degradation of HCHO by hydrogen peroxide. The present state of affairs indicates that reaction systems employing hydrogen peroxide as an oxidant, photocatalytic systems, and electrocatalytic systems demonstrate inadequate degradation efficiency for HCHO (Table 1).

Although the catalytic activity of lanthanum cobalt oxide for HCHO degradation decreases with an increase in the La/Co molar ratio, its catalytic activity for PMS decomposition increases with an increase in the La/Co molar ratio. However, the catalytic effect of lanthanum oxide on PMS decomposition is extremely weak, and the structure and composition of lanthanum cobalt oxides still warrant in-depth investigation.

## 5. Conclusions

When the concentration of HCHO was 1.086 mg/mL (5 mL), the dosage of LaCoO_3_-1 was 8 mg/mL, and n(PMS)/n(HCHO) = 2.5, the removal rate of HCHO exceeded 90% across a pH range of 3 to 11 after 10 min at 25 °C. A comparison of the present report with previous reports concerning the catalytic oxidative degradation of HCHO in water (Table 1) reveals the superior catalytic activity of the perovskite-type LaCoO_3_-activated PMS system for HCHO degradation at room temperature. This reaction occurs without the need for light or electricity and results in a reduced reaction time.

XRD, XPS and TEM analysis confirmed the formation of perovskite structure. The catalytic HCHO degradation process of perovskite-type LaCoO_3_ is significantly different from that of La_2_O_3_ and Co_2_O_3_. The cooperation between La and Co significantly increased the degradation of HCHO. The complete degradation of HCHO in the research depended on the Co^2+^ on the catalyst surface. The introduction of La_2_O_3_ significantly increased the oxygen vacancy and Co^2+^ content.

Unfortunately, LaCoO_3_ can only catalyze the decomposition of hydrogen peroxide, but cannot provide free radicals for reaction with HCHO. This limits the use of hydrogen peroxide as a green oxidant.

## Figures and Tables

**Table 1:** Comparison of the catalytic oxidative degradation of HCHO in water.

Catalyst	Degradation (%)	Oxidant	Temperature (℃)	Reaction Time (min)	Reference
MIL-100(Fe)	93	H_2_O_2_/visible 132 radiation (Xenon, 55 W)	Ambient temperature	119	[50]
MgO	79	H_2_O_2_/O_3_	Ambient temperature	120	[51]
-	91	H_2_O_2_/UV	Ambient temperature	210	[52]
Bi_2_MoO_6_/attapulgite	58	150 W visible light	Ambient temperature	120	[53]
-	85	Vacuum UV	Ambient temperature	60	[54]
-	69	ferrate (VI)	25	35	[55]
-	17.5	Solar Thermal Electrochemical	25	60	[56]

## Data Availability

The original contributions presented in this study are included in the article. Further inquiries can be directed to the corresponding author.
